# Decades of discovery: unveiling emerging trends, pivotal research areas, and landmark publications in national tobacco research in India

**DOI:** 10.3389/frma.2025.1496571

**Published:** 2025-07-03

**Authors:** Alok Singh, Akanksha Kashyap, Saurabh Varshney, Sudip Bhattacharya

**Affiliations:** ^1^Faculty of Medicine and Health Sciences, Shree Guru Gobind Singh Tricentenary University, Gurugram, Haryana, India; ^2^Mahatma Gandhi Kashi Vidyapith, Varanasi, Uttar Pradesh, India; ^3^All India Institute of Medical Sciences, Deoghar (AIIMS Deoghar), Deoghar, India

**Keywords:** tobacco research, research gaps, GATS, GYTS, India, NTCP

## Abstract

**Introduction:**

Tobacco use remains a major public health concern in India, contributing significantly to the burden of non-communicable diseases and premature mortality. Over the past two decades, national tobacco research has evolved in response to shifting regulatory frameworks, scientific developments, and increasing awareness of tobacco's health, social, and environmental implications. The World Health Organization Framework Convention on Tobacco Control (WHO FCTC), adopted in 2003, has played a catalytic role in aligning research efforts with global priorities. However, there is a need to map the growth and direction of this research to identify strengths, gaps, and emerging trends within the Indian context.

**Methods:**

A bibliometric analysis was conducted to evaluate tobacco-related research output affiliated with Indian institutions between 2003 and 2024. Data were retrieved from the SCOPUS database, limited to peer-reviewed journal articles and reviews published in English. Analytical tools included SCOPUS Analytics, Microsoft Excel, the Biblioshiny package in R, and VOS viewer software. These tools were used to extract and visualize trends in publication volume, subject categories, key authors, institutional collaborations, citation metrics, and thematic hotspots. Inclusion criteria were confined to Indian-affiliated institutions contributing to national and global discourse on tobacco research.

**Results:**

Tobacco research in India showed a consistent upward trend post-2003, with notable surges corresponding to key public health developments. The majority of publications were concentrated in the domain of medicine, particularly focusing on cancer prevention and tobacco control interventions. Leading contributors included institutions such as the All India Institute of Medical Sciences (AIIMS), with significant collaborations observed with United States-based researchers. Despite increased output, research on the social and environmental consequences of tobacco use remained limited. Additionally, international collaboration was relatively low, and funding was primarily from Indian agencies, with minimal support from global or low-income country partnerships. Notably, recent studies employed advanced methodologies, such as machine learning and nanotechnology, and examined newer themes like the intersection of tobacco use and COVID-19-related respiratory risks.

**Discussion:**

The findings indicate a maturing research ecosystem around tobacco in India, strongly anchored in biomedical science and policy interventions. However, the underrepresentation of interdisciplinary studies exploring socio-cultural and ecological dimensions suggests a critical gap. Moreover, the low levels of international collaboration and inadequate funding for low-income contexts highlight systemic limitations that could hinder progress. To advance tobacco research nationally and globally, enhanced collaboration across disciplines and geographies is essential. Incorporating emerging technologies and focusing on equity-driven research agendas will be pivotal in addressing the multifaceted impact of tobacco use.

## Introduction

National tobacco research is undergoing significant transformation as public health concerns, regulatory pressures, and scientific advancements shape the field (van der Eijk, 2015). Emerging trends reflect a growing emphasis on tobacco control, harm reduction, and the effects of new nicotine delivery systems such as e-cigarettes and heated tobacco products (O'Connor et al., 2022). Key research areas now focus on the health impacts of tobacco use, the social and economic factors influencing smoking behavior, and effective cessation strategies. Environmental tobacco smoke, youth-targeted marketing, and national disparities in tobacco-related harm are also critical areas of inquiry. Influential publications in this domain, such as “Tobacco Control,” “Nicotine and Tobacco Research,” and reports from the World Health Organization (WHO), provide cutting-edge insights, guiding policy and public health interventions (Joseph et al., 2021). As tobacco use remains a leading cause of preventable deaths nationally, ongoing research is crucial for shaping effective, evidence-based responses to this pressing health challenge (John, 2005; Mishra et al., 2012; Services et al., 2014; Nazar et al., 2020; Shaikh et al., 2022; Pahari et al., 2023). Research in national tobacco control is essential because tobacco use remains one of the leading causes of preventable illness and deaths worldwide. Understanding emerging trends, such as the rise of e-cigarettes and novel nicotine products, is critical for addressing their potential health impacts and informing public policy (Tattan-Birch et al., 2024). Key research areas, including the social determinants of tobacco use, youth smoking prevention, and effective cessation strategies, help in developing interventions that can reduce the national burden of tobacco-related diseases. Moreover, influential publications and studies drive evidence-based decision-making, shaping regulations, public health campaigns, and international frameworks, such as the World Health Organization's Framework Convention on Tobacco Control (FCTC; Myers et al., 2023). By continuously advancing tobacco research, we can mitigate the immense social, economic, and health costs associated with smoking and nicotine addiction (KA et al., 1996; Warner, 2005; Lindson et al., 2017; Berg et al., 2018; Meissner et al., 2022). Despite decades of tobacco control efforts, significant gaps persist in our understanding of the evolving landscape of nicotine consumption, particularly concerning the long-term health consequences of emerging products such as e-cigarettes and heated tobacco devices. These products are often introduced with claims of harm reduction, yet empirical data on their safety profiles and potential to facilitate cessation remain inconclusive. Simultaneously, socio-cultural drivers of tobacco use in low- and middle-income countries, including India, are underexplored in the literature, despite these regions bearing a disproportionate burden of tobacco-related morbidity. This bibliometric study addresses these critical voids by mapping national tobacco research trends from 2003 to 2024, thereby highlighting not only the scientific focus on biomedical impacts but also the relative neglect of behavioral, cultural, and socio-economic determinants (Salt and Osborne, 2020). Through a systematic analysis of publication patterns, research clusters, and funding sources, the study provides an evidence-based framework for identifying overlooked areas and guiding future interdisciplinary research and policy interventions (Ribisl, 2012; Hicks et al., 2019; Claire et al., 2020; Parascandola et al., 2022; Puljević et al., 2022).

### Aim

The aim of this study was to conduct a comprehensive analysis of national tobacco research from 2003 to 2024, with a focus on identifying emerging trends, examining key research areas, and evaluating influential publications.

### Objectives

Our study objectives were:

1. To identify and analyse emerging trends in tobacco use and control, including the rise of novel nicotine delivery systems such as e-cigarettes, heated tobacco products, and vaping, and their implications for public health.
2. To examine key research areas that have shaped the field, such as the health impacts of tobacco products, cessation strategies, regulatory frameworks, and the socio-economic factors influencing tobacco use.
3. To evaluate the most influential publications in tobacco research, including peer-reviewed articles, reports, and policy documents, to understand their role in shaping national tobacco control strategies and public health policies.

## Methodology

### Pre-planning

**Figure 1:**
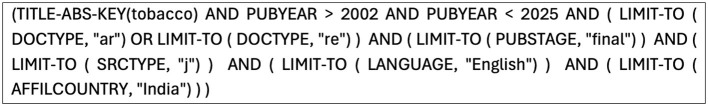
List of keywords.

During the pre-planning phase, search queries were identified and are presented in Figure 1. These queries were related to tobacco research and India. By including criteria for tobacco with terms India, our goal was to investigate the fundamental, intellectual and conceptual landscape of studies exploring the tobacco research in India from 2003 to 2024. This data is crucial for understanding tobacco research in India. Moreover, specific research questions, outlined in Table 1, were formulated to provide a comprehensive overview of the knowledge structure and the bibliometric and statistical methods used to evaluate tobacco research from 2003 to 2024.

**Table 1:** Integration of research questions with the knowledge structure using bibliometric and statistical methodologies.

**SL No**	**Research questions**	**Knowledge structure covered**	**Bibliometric techniques**
1.	What are the publishing trends of the research publication in tobacco research?	Intellectual structure	Annual scientific production
2.	Who are the most contributing authors, journals, organizations, funding agencies, and countries, and cited papers in tobacco research?	Intellectual structure	Analysis of most prolific authors, journal, organization, funding agencies, countries and top cited articles
3.	What are the publication patterns and most frequently used keywords of the articles published in tobacco research?	Conceptual structure	Annual scientific production, co-citation of references, authors keyword occurrence and authors keyword co-occurrence analysis
4.	What are the trending topics of the tobacco research?	Conceptual structure	Analysis of trending topics, authors keyword occurrence analysis and authors keyword co-occurrence analysis
5.	What are the main open areas of challenges and the corresponding solutions for future research work in tobacco research?	Conceptual structure	Analysis of trending topics, authors keyword occurrence analysis and authors keyword co-occurrence analysis

### Data collection

During the data collection process, a systematic search was conducted in the SCOPUS core collection to collect articles published from January 1, 2003, to September 11, 2024, with a focus on tobacco research in India. The search terms used for retrieving data are detailed in Figure 1. Only research articles and review papers published in English were included. This process identified a total of 9,815 publications in SCOPUS for analysis after screening.

### Data extraction

Metadata were retrieved from the Scopus database in CSV format, encompassing comprehensive bibliographic information. The dataset included the following elements: (a) names of authors and editors, (b) complete author names, (c) article titles, (d) publication sources, (e) author-supplied keywords, (f) indexed keywords, (g) abstracts, (h) institutional affiliations of authors, (i) corresponding authors' affiliations, (j) cited references, (k) citation counts, (l) records of highly cited publications, (m) usage statistics, (n) year of publication, (o) digital object identifiers (DOIs), (p) subject classifications, (q) unique author identification codes, (r) publication languages, and (s) funding bodies.

### Data refinement

**Figure 2:**
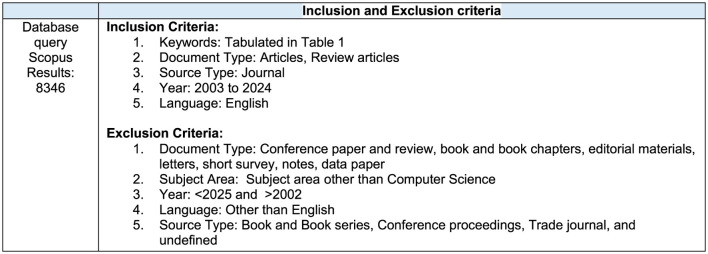
Eligibility criteria for selection of study.

**Figure 3:**
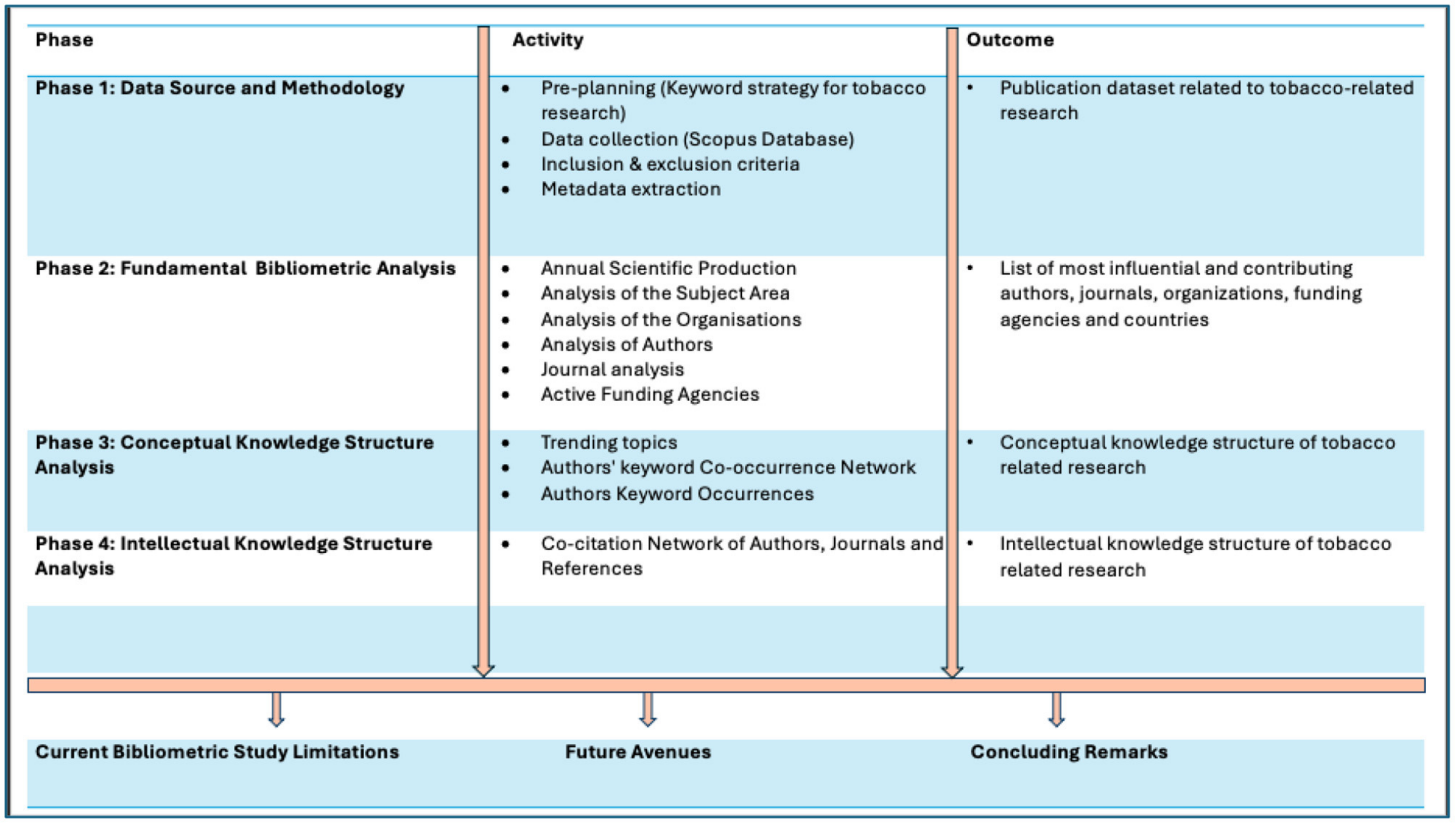
Different phases of bibliometric analysis of scientific literature on ultra-processed foods in cancer research from 2003 to 2024.

**Figure 4:**
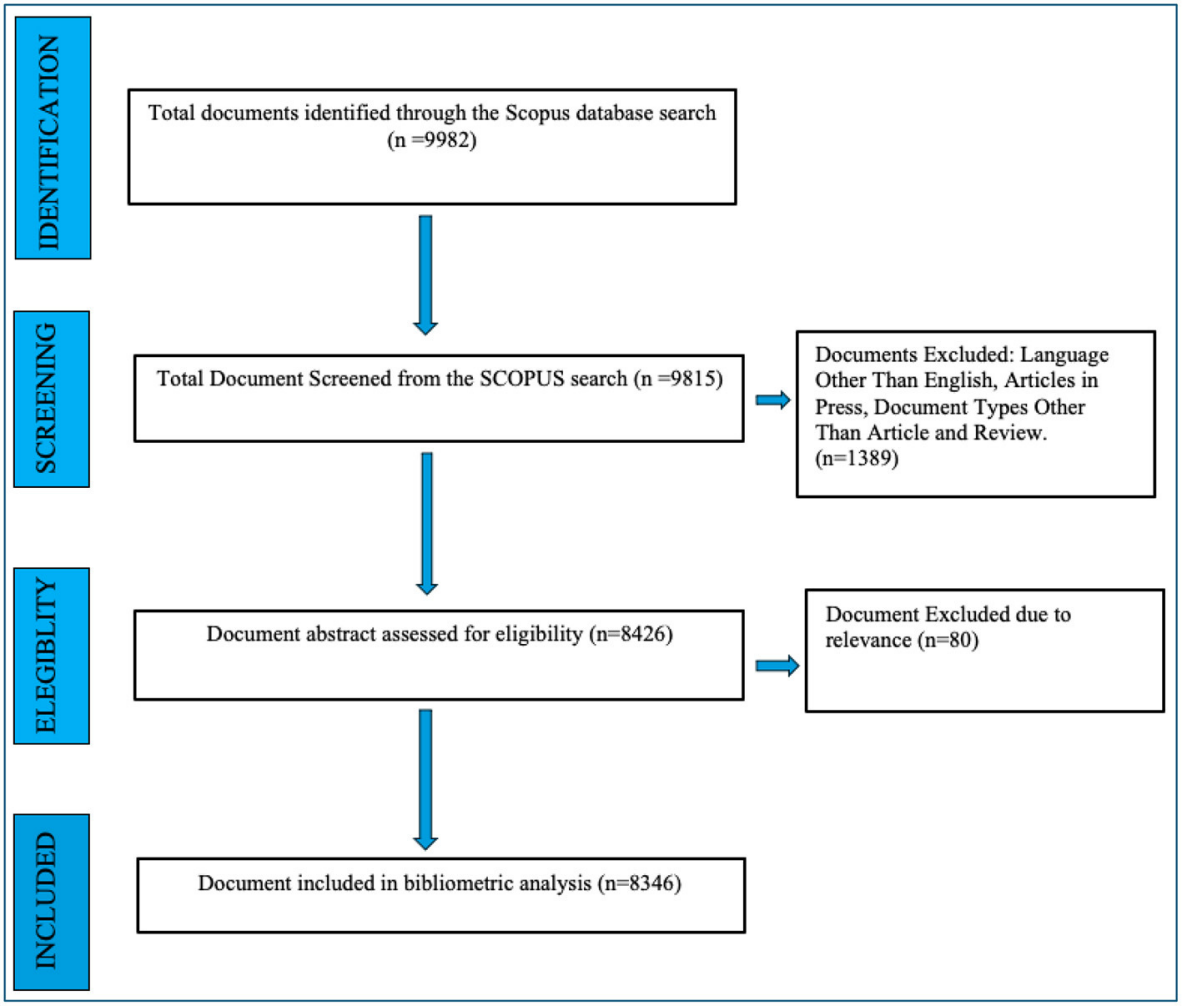
Results of the keywords search as per PRISMA guidelines.

In the data refinement stage, the initial dataset comprising 9,982 publications retrieved from the Scopus database was screened and filtered based on predefined exclusion criteria, as outlined in Figure 2. Publications categorized as books, editorials, letters, conference proceedings, or non-peer-reviewed materials were excluded. Additionally, studies not published in the English language were removed to maintain consistency in content analysis. The screening process was independently conducted by two reviewers (A.S and SV) to ensure objectivity and methodological rigor. Discrepancies or conflicts regarding study inclusion were addressed through consultation with a third reviewer (SB), employing a consensus-driven methodology to reach a resolution. Following the application of these exclusion criteria, the final dataset comprised 8,346 peer-reviewed articles relevant to tobacco research. A systematic overview of the selection and refinement process is presented in Figures 2–4.

### Bibliometric analysis

Bibliometric analysis serves as a robust and systematic method for organizing, accessing, and evaluating large volumes of scientific literature. It facilitates a comprehensive understanding of evolving research trends within a specific domain. (4) In the present study, a bibliometric analysis was conducted on publications related to tobacco research from 2003 to 2024, guided by the five key research questions outlined in Table 1. For the analytical process, the tools Biblioshiny and VOSviewer were employed to assess publication dynamics and research patterns in this field. Furthermore, this analysis seeks to map and evaluate the intellectual structure and development of scientific knowledge pertaining to tobacco research. The foundational knowledge base within this research area is conceptualized into two primary components:

#### Conceptual structure

Examines the principal themes and emerging research trends that define the intellectual landscape of a specific scholarly domain.

#### Intellectual structure

Evaluates the scholarly impact and significance of individual authors based on their contributions to the advancement of knowledge within the academic community.

The conceptual structure is analyzed through statistical methods such as the Authors' keyword co-occurrence network analysis, the Authors' keywords occurrence and trending topics. Following this, the intellectual structure is assessed using co-citation network analysis. By examining these two dimensions—conceptual, intellectual this study seeks to understand the knowledge framework underpinning tobacco research from 2003 to 2024. (6) This analysis aims to showcase key accomplishments and identify future research directions in the field.

## Results

### Fundamental bibliometric analysis

#### Annual publications and trends

**Figure 5:**
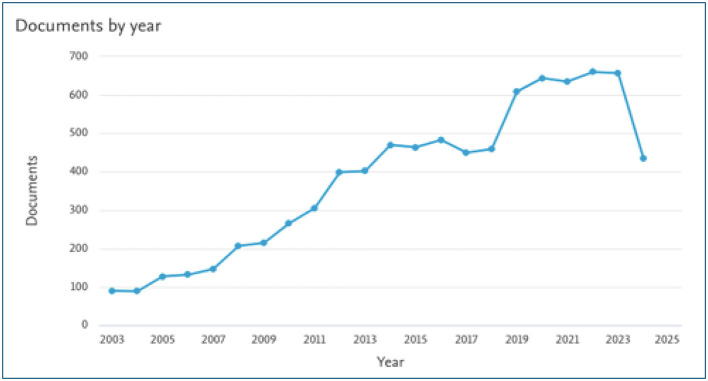
Annual scientific production on tobacco research in India (2003–2024; source: Scopus).

The data presented in Figure 5 illustrates a general upward trend in tobacco-related publications in India over the past two decades. Until 2007, the annual number of publications remained below 200. However, a consistent year-on-year increase was observed after 2007, indicating that tobacco research in India entered a phase of rapid development. The highest output was recorded in 2023, with 657 publications in that year.

#### Analysis of the subject area

**Figure 6:**
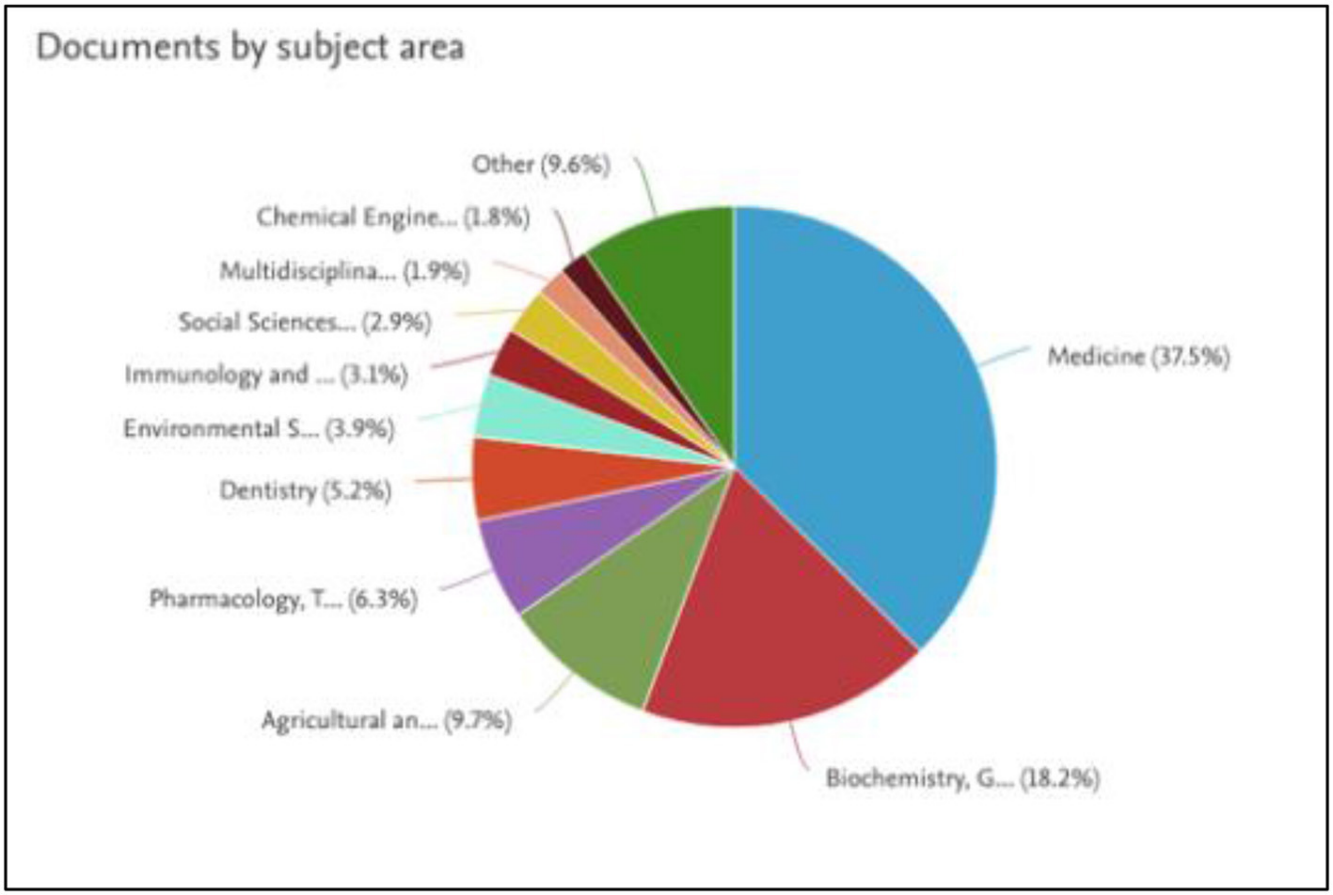
Distribution of tobacco-related publications by academic subject area (source: Scopus).

The pie chart in Figure 6 presents the distribution of research output on tobacco across different subject areas. The majority of studies are concentrated in the field of Medicine, comprising 37%. This is followed by Biochemistry, Genetics, and Molecular Biology at 18.2%, Agricultural and Biological Sciences at 9.7%, and Pharmacology, Toxicology, and Pharmaceutics at 6.3%. Other disciplines include Dentistry (5.2%), Environmental Science (3.9%), Immunology and Microbiology (3.1%), Social Sciences (2.9%), Multidisciplinary research (1.9%), and Chemical Engineering (1.8%), with 9.6% attributed to various other fields. This analysis highlights Medicine as the primary focus of tobacco-related research.

#### Analysis of the organizations

**Figure 7:**
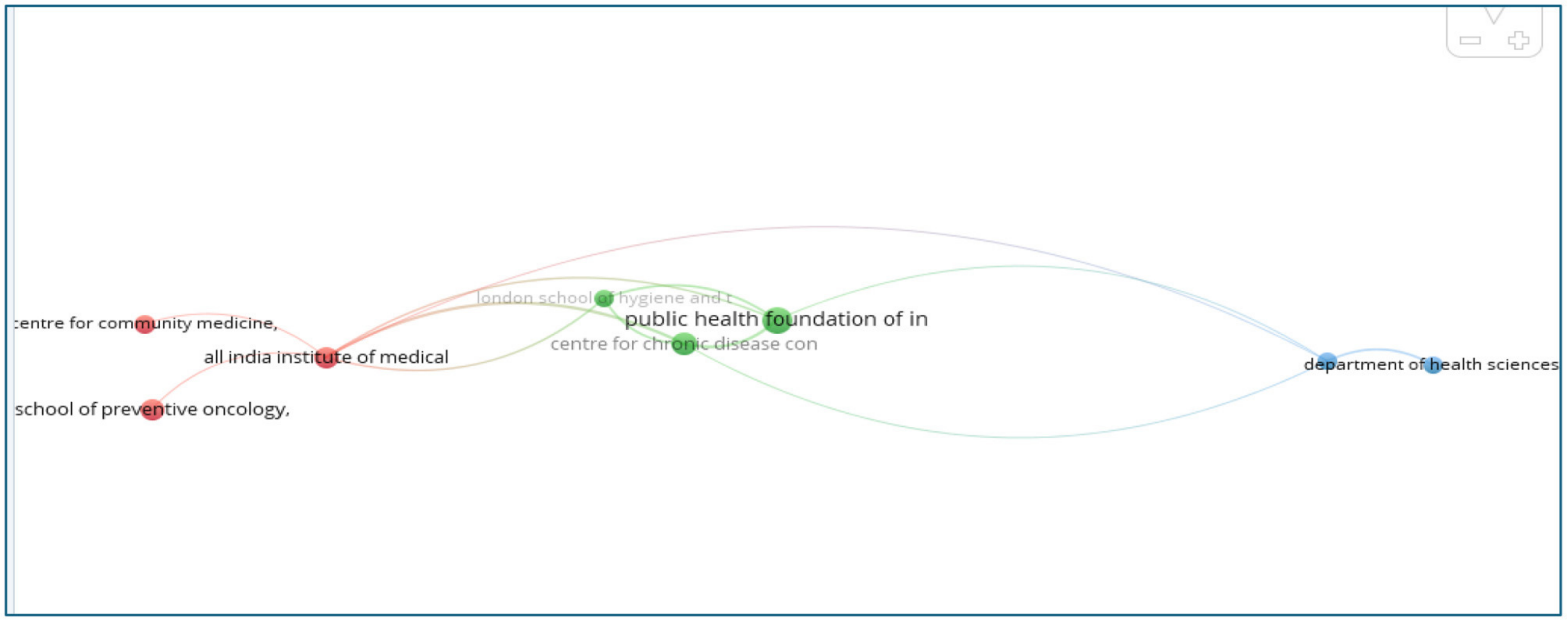
Network visualization of institutional co-authorship in tobacco research in India (2003–2024).

The study analyzed contributions from 16,793 distinct organizations, with 10 institutions publishing at least 147 papers. The All India Institute of Medical Sciences, New Delhi, ranked highest with 359 papers, followed by the Postgraduate Institute of Medical Education and Research, Chandigarh (283 papers), and the Public Health Foundation of India (253 papers). Other significant contributors included Saveetha Dental College and Hospitals with 240 papers, the ICMR (Indian Council of Medical Research) with 226, and the Saveetha Institute of Medical and Technical Sciences with 225 papers (Table 2). VOS viewer software was employed to visualize co-authorship between institutions, helping to identify collaborative networks and track their evolution over time. The analysis focused on institutions that produced 10 or more papers, with the results presented in Figure 7. In this visualization, node size represented publication volume, links depicted co-authorship connections, and node colors indicated distinct clusters. From the 16,793 organizations, 18 institutions were included in the analysis. The most productive institution, All India Institute of Medical Sciences, New Delhi (Cluster 1, red), is strongly connected with the Center for Community Medicine, New Delhi, and the School of Preventive Oncology, Patna. Cluster 2 (green) featured the Center for Chronic Disease, New Delhi, collaborating closely with the London School of Hygiene and Tropical Medicine, UK, and the Public Health Foundation of India. Cluster 3 (blue) involved the Department of Health Sciences, University of York, UK, and Hriday, Delhi.

**Table 2:** Leading Indian institutions in tobacco research (2003–2024).

**Organizations**	**Documents**
All India Institute of Medical Sciences, New Delhi	359
Postgraduate Institute of Medical Education and Research, Chandigarh	283
Public Health Foundation of India	253
Saveetha Dental College and Hospitals	240
Indian Council of Medical Research	226
Saveetha Institute of Medical and Technical Sciences	225
Manipal Academy of Higher Education	199
Tata Memorial Hospital	177
King George's Medical University	164
University of Delhi	147

The findings suggest that inter-institutional collaborations predominantly occur within national borders. Institutions with higher publication outputs tended to collaborate more frequently with others, indicating that fostering institutional partnerships could enhance the quality and quantity of research outputs.

#### Analysis of authors

**Figure 8:**
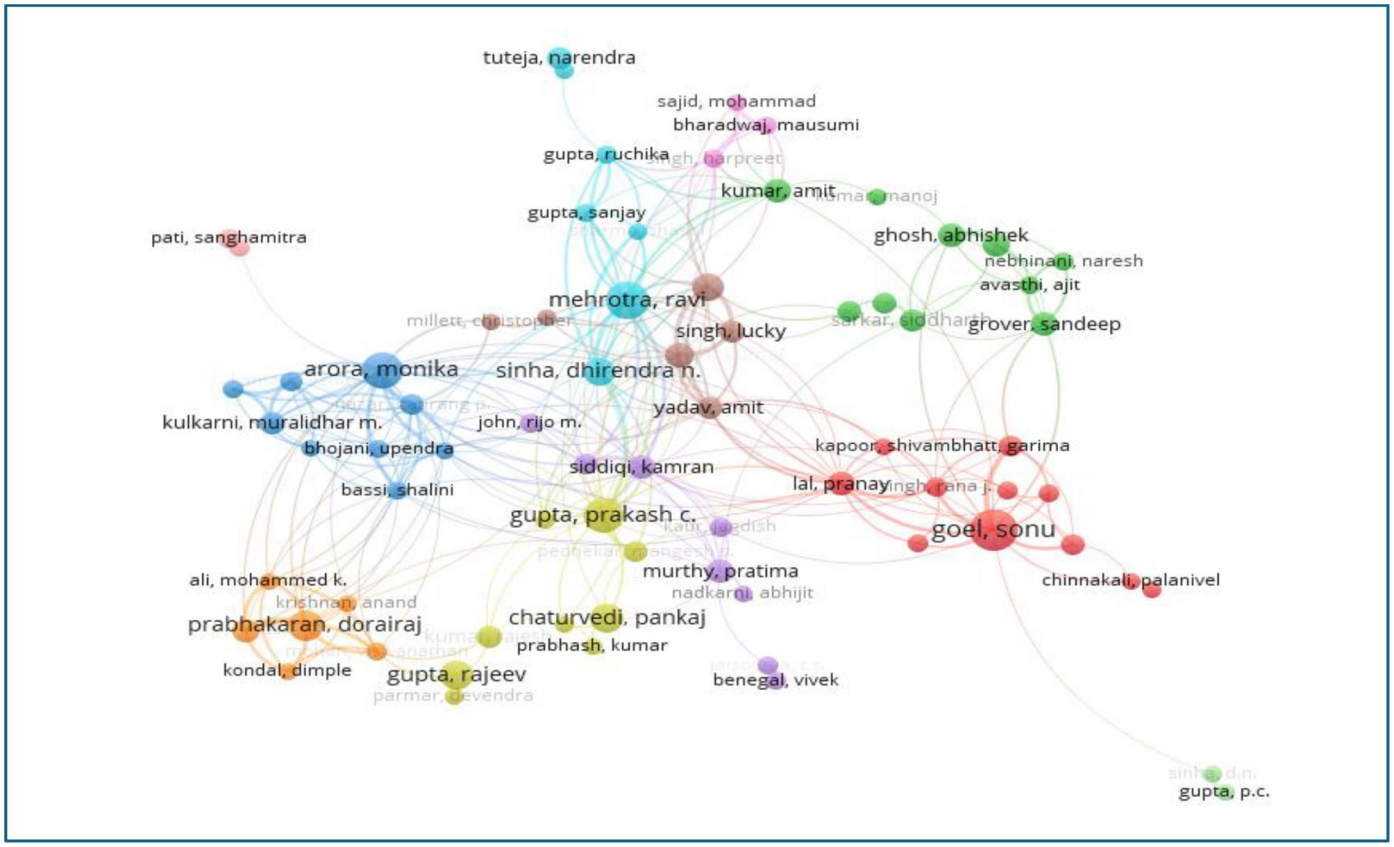
Network visualization map showing co-authorship relationships among the most prolific authors in Indian tobacco research.

A total of 21,934 researchers participated in tobacco-related studies, with 10 authors contributing 53 or more papers each. Table 3 highlights these prolific authors, who collectively produced 797 publications, accounting for 9.54% of total submissions. Among them, Gupta, Prakash Chandra from the Healis Sekhsaria Institute for Public Health, Navi Mumbai, was the most productive, publishing 140 papers. He was followed by Sinha, Dhirendra Narain (114 papers), also from Healis Sekhsaria, and Arora, Monika (97 papers) from the Public Health Foundation of India, New Delhi. The research work by Lim, Stephen S. and colleagues had the highest citation count, with 9,410 citations. The top 10 authors were primarily based in India, USA. Based on Price's Law, the equation m = 0.749 × √(max(n)) was applied, where ‘m' represents the minimum number of publications by leading authors, and “max(n)” is the total number of publications by the most prolific author. Authors with 11 or more publications were categorized as core contributors. VOS viewer software was employed to visualize co-authorship networks, which track the development of research collaborations over time. Each “bubble” in the visualization represents an author, with its size indicating the number of publications. Lines connecting authors denote co-authorship links, and authors close to each other on the map have frequently co-authored papers. Different colors represent clusters of authors with strong co-authorship connections. Of the 21,934 authors with 11 or more publications, 107 met the threshold and were divided into 11 clusters (Figure 8).

**Table 3:** Leading authors in tobacco research in India (2003–2024).

**Author name**	**Country**	**Documents**
Gupta, P.C.	India	140
Sinha, D.N.	India	114
Arora, M.	India	97
Mehrotra, R.	United States	74
Goel, S.	India	69
Gupta, R.	India	67
Pednekar, M.S.	India	65
Prabhakaran, D.	India	62
Reddy, K.S.	India	56
Chaturvedi, P.	India	53
Total		797

#### Journal analysis

**Figure 9:**
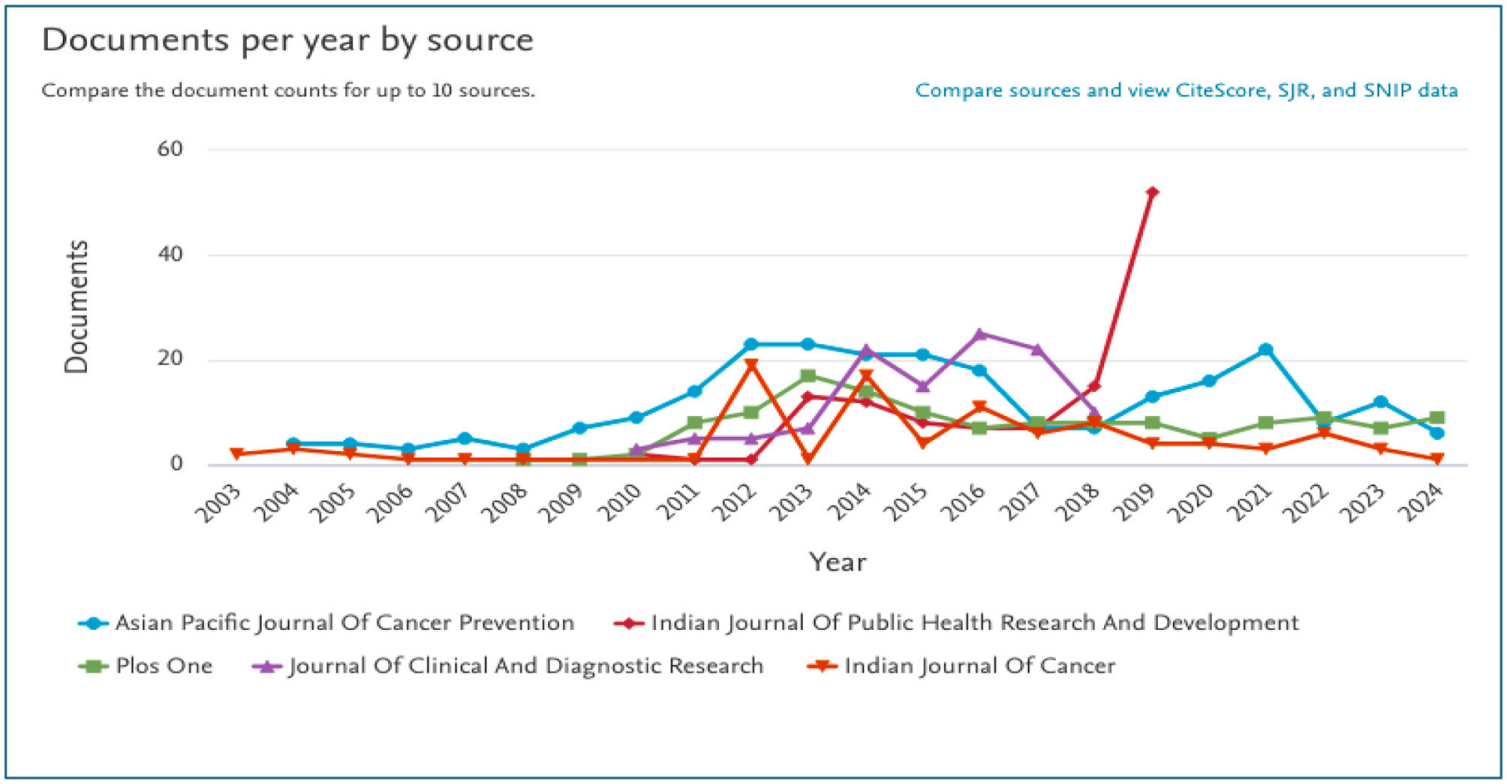
Annual distribution of publications by leading journals.

A total of 1,796 academic journals have published research articles on tobacco. Of these, the 10 most active journals accounted for 1,117 out of 8,346 papers, contributing 62.19% of the total publications (Table 4). The *Asian Pacific Journal of Cancer Prevention* led with 246 articles, followed by *PLoS ONE* (132), the Indian Journal of Public Health Research and Development (118), and the *Journal of Clinical and Diagnostic Research* (114). Other significant contributors were *Indian Journal of Cancer* (98), *Indian Journal of Community Medicine* (93), *Journal of Cancer Research and Therapeutics* (83), *Indian Journal of Public Health* (79), *Indian Journal of Medical Research* (78), and *Indian Journal of Psychiatry* (76) (Table 3). The *Indian Journal of Public Health Research and Development* has been publishing on tobacco since before 2003, with a peak in 2012. The *Asian Pacific Journal of Cancer Prevention*, which began in 2004, was the leading publisher on this topic until 2011, after which it lost its dominance. Between 2011 and 2018, the *Journal of Clinical and Diagnostic Research* and the *Indian Journal of Public Health Research and Development* became the top publishers in the field (Figure 9).

**Table 4:** Top journals publishing tobacco research in India (2003–2024).

**Source title**	**IF**	**Country**	**Documents**
Asian Pacific Journal of Cancer Prevention	91	Thailand	246
PLoS ONE	435	United States	132
Indian Journal of Public Health Research and Development	23	India	118
Journal of Clinical and Diagnostic Research	72	India	114
Indian Journal of Cancer	44	India	98
Indian Journal of Community Medicine	43	India	93
Journal of Cancer Research and Therapeutics	51	India	83
Indian Journal of Public Health	35	India	79
Indian Journal of Medical Research	104	India	78
Indian Journal of Psychiatry	53	India	76
Total			1,117

#### Active funding agencies

Of the 8,346 articles reviewed, 1,940 were funded by the top 10 organizations. The Council of Scientific and Industrial Research (CSIR), India, was the largest contributor, backing 301 tobacco-related studies. Other major Indian funders included the Department of Biotechnology (*n* = 299), the Department of Science and Technology (*n* = 263), the University Grants Commission (*n* = 247), the Indian Council of Medical Research (*n* = 43), and the Science and Engineering Research Board (*n* = 89). Internationally, the National Institutes of Health (*n* = 174) and the U.S. Department of Health and Human Services (*n* = 111) were prominent contributors, along with the Bangladesh Council of Scientific and Industrial Research (*n* = 96). Seven of the top 10 funders were from India, with two from the USA and one from Bangladesh (Table 5).

**Table 5:** Top funding bodies supporting tobacco research in India (2003–2024).

**Top funding agency**	**Country**	**Documents**
Council of Scientific and Industrial Research	India	301
Department of Biotechnology, Ministry of Science and Technology	India	299
Department of Science and Technology, Ministry of Science and Technology	India	263
Indian Council of Medical Research	India	255
University Grants Commission	India	247
National Institutes of Health	The United States	174
U.S. Department of Health and Human Services	The United States	111
Department of Biotechnology, Government of West Bengal	India	105
Bangladesh Council of Scientific and Industrial Research	Bangladesh	96
Science and Engineering Research Board	India	89
Total		1,940

### Analysis of intellectual knowledge structure

#### Analysis of co-cited journals

**Figure 10:**
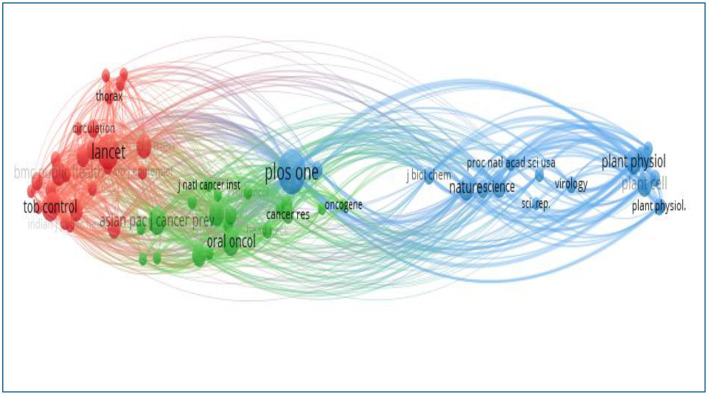
Network visualization of co-cited journals.

Table 6 presents the 10 journals with the highest citation counts. A journal's impact within its field is reflected by the frequency of its co-citations, serving as an indicator of its importance. Remarkably, two of the journals listed in Table 5 have been cited more than 2,000 times. Figure 10 illustrates that the three most frequently co-cited journals are *PLoS ONE* (Q1, H-index: 435), *The Lancet* (Q1, H-index: 895), and *Oral Oncology* (Q1, H-index: 137).

**Table 6:** Top 10 co-cited journals in tobacco research (2003–2024).

**Source**	**Citations**	**Total link strength**	**Quartile**
PLoS ONE	3,408	35,611	Q1
The Lancet	2,050	21,339	Q1
Oral Oncology	1,332	18,144	Q1
Tobacco Control	1,311	12,372	Q1
Asian Pacific Journal of Cancer Prevention	1,274	13,830	Q3
Plant Physiology	1,066	16,620	Q1
Plant Cell Reports	1,039	17,566	Q1
International Journal Of Cancer	992	16,718	Q1
BMC Public Health	981	9,100	Q1
The New England Journal Of Medicine	960	13,417	-
Nature	958	12,550	Q1

#### Analysis of co-cited authors

**Figure 11:**
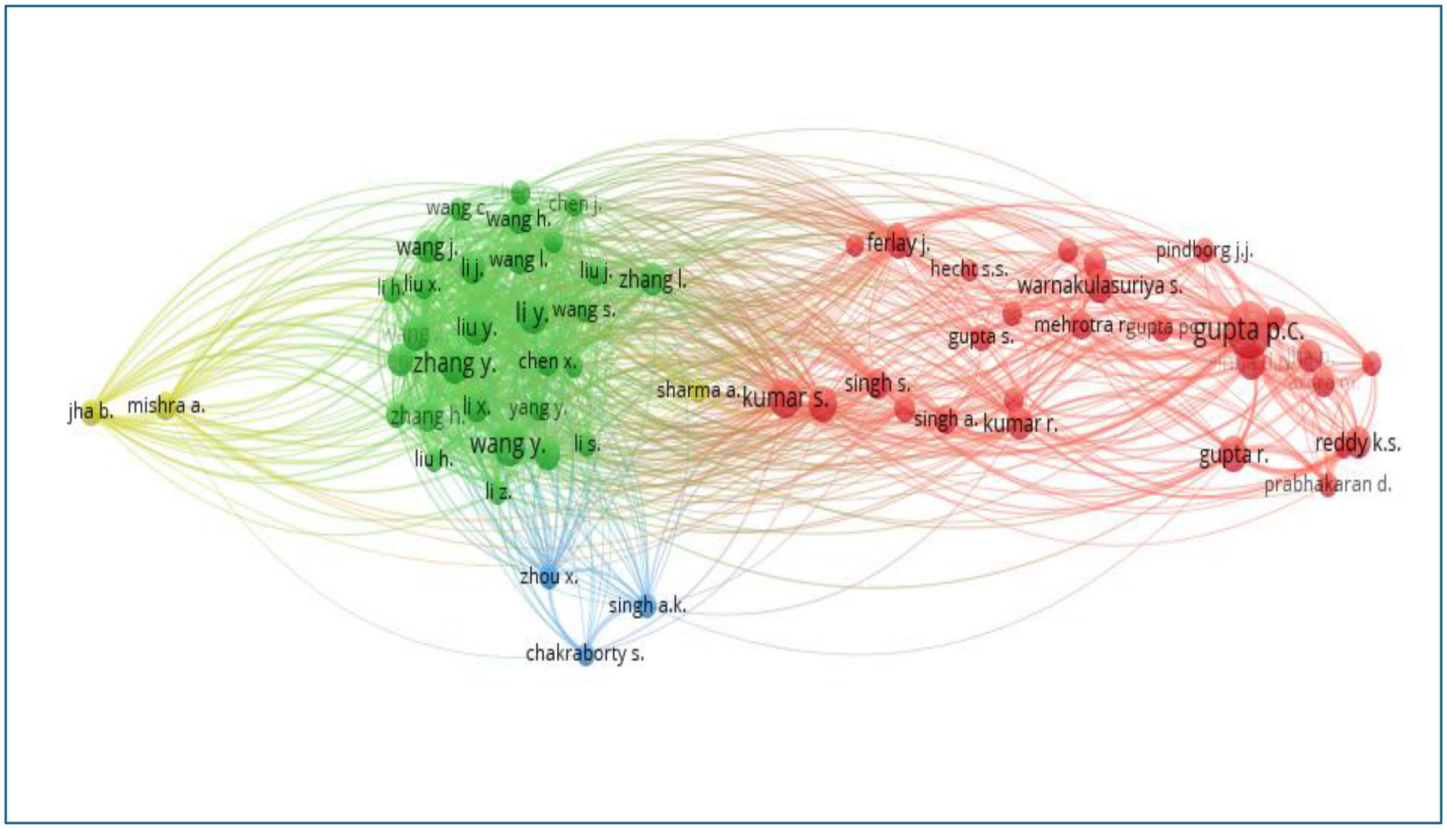
Network visualization of co-cited authors in tobacco research (2003–2024).

The co-cited authors in the literature are visualized in Figure 11 using VOS viewer. Co-citation relationships reflect the connection between authors whose works are jointly referenced in other articles. The size of each node in the graph represents the number of citations an author has received, with larger nodes corresponding to higher citation counts and greater influence. Gupta PC leads with 1,593 co-citations, followed by Wang Y, Kumar S, and Zhang Y, with 885, 796, and 732 co-citations, respectively (Table 7). The top 10 co-cited authors amassed over 7,995 co-citations, underscoring their significant impact on tobacco research.

**Table 7:** Most frequently co-cited authors in Indian tobacco research (2003–2024).

**Sl. No**	**Author**	**Co-citations**
1.	Gupta, P.C.	1,593
2.	Wang Y.	885
3.	Kumar S.	796
4.	Zang Y.	732
5.	Li Y	701
6.	Liu Y.	690
7.	Sinha D.N	675
8.	Gupta R.	674
9.	Kumar A.	643
10.	Wang X.	606
Total co citations		7,995

#### Analysis of co-cited references

**Figure 12:**
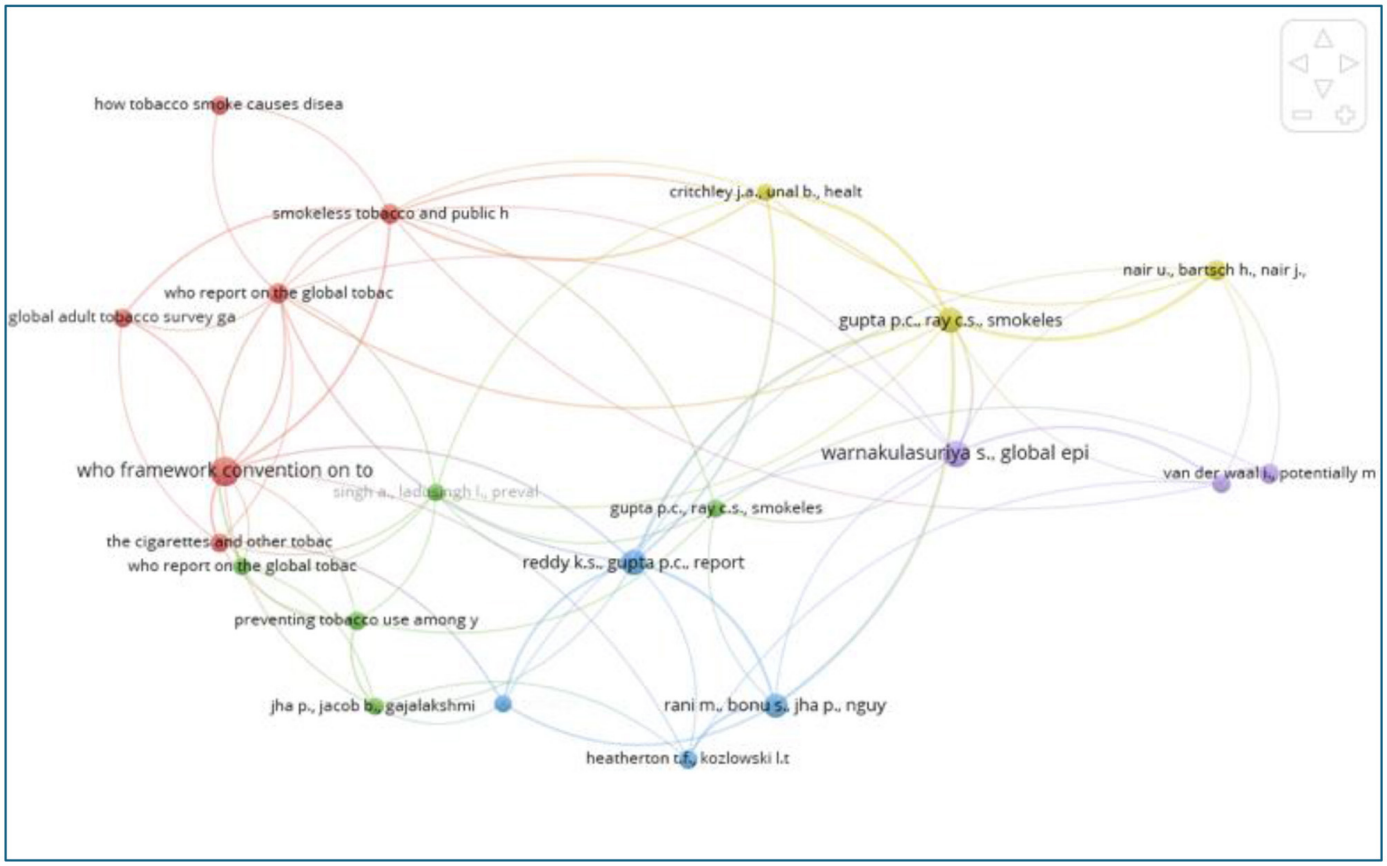
The network visualization map of co-cited references.

Co-citation analysis identifies references that are frequently cited together by other works, serving as a widely used method for investigating research trends in a specific academic discipline. The co-citation network is illustrated in Figure 12, while Table 8 presents the top 10 most-cited articles. Our analysis reveals that these 10 articles primarily focus on three main research themes. (1) tobacco control, (2) tobacco and cancer, (3) prevalence and predictors of tobacco.

**Table 8:** Network visualization of the top 10 most frequently co-cited references in Indian tobacco research (2003–2024).

**Sl. No**	**Cited article**	**Citations**	**Total link strength**
1.	WHO Framework Convention on Tobacco Control (WHO FCTC; Myers et al., 2023)	55	16
2.	National epidemiology of oral and oropharyngeal cancer (Warnakulasuriya, 2009)	47	13
3.	Report on Tobacco Control in India (Chengappa et al., 2024)	44	23
4.	Smokeless tobacco and health in India and South Asia (Gupta and Ray, 2003)	42	33
5.	Tobacco use in India: prevalence and predictors of smoking and chewing in a national cross sectional household survey (Rani et al., 2003)	38	16
6.	A Simple and General Method for Transferring Genes into Plants (Horsch et al., 1985)	30	10
7.	A rapid and sensitive method for the quantitation of microgram quantities of protein utilizing the principle of protein-dye binding (MM, 1976)	29	6
8.	WHO report on the national tobacco epidemic, 2008: the MPOWER package (Zhang et al., 2022)	28	15
9.	Alert for an epidemic of oral cancer due to use of the betel quid substitutes gutkha and pan masala: a review of agents and causative mechanisms (Nair et al., 2004)	28	13
10.	The Use of DNA Extraction for Molecular Biology and Biotechnology Training: A Practical and Alternative Approach (Lázaro-Silva et al., 2015)	28	6

### Conceptual knowledge structure analysis

#### Analysis of keyword co-occurrence

**Figure 13:**
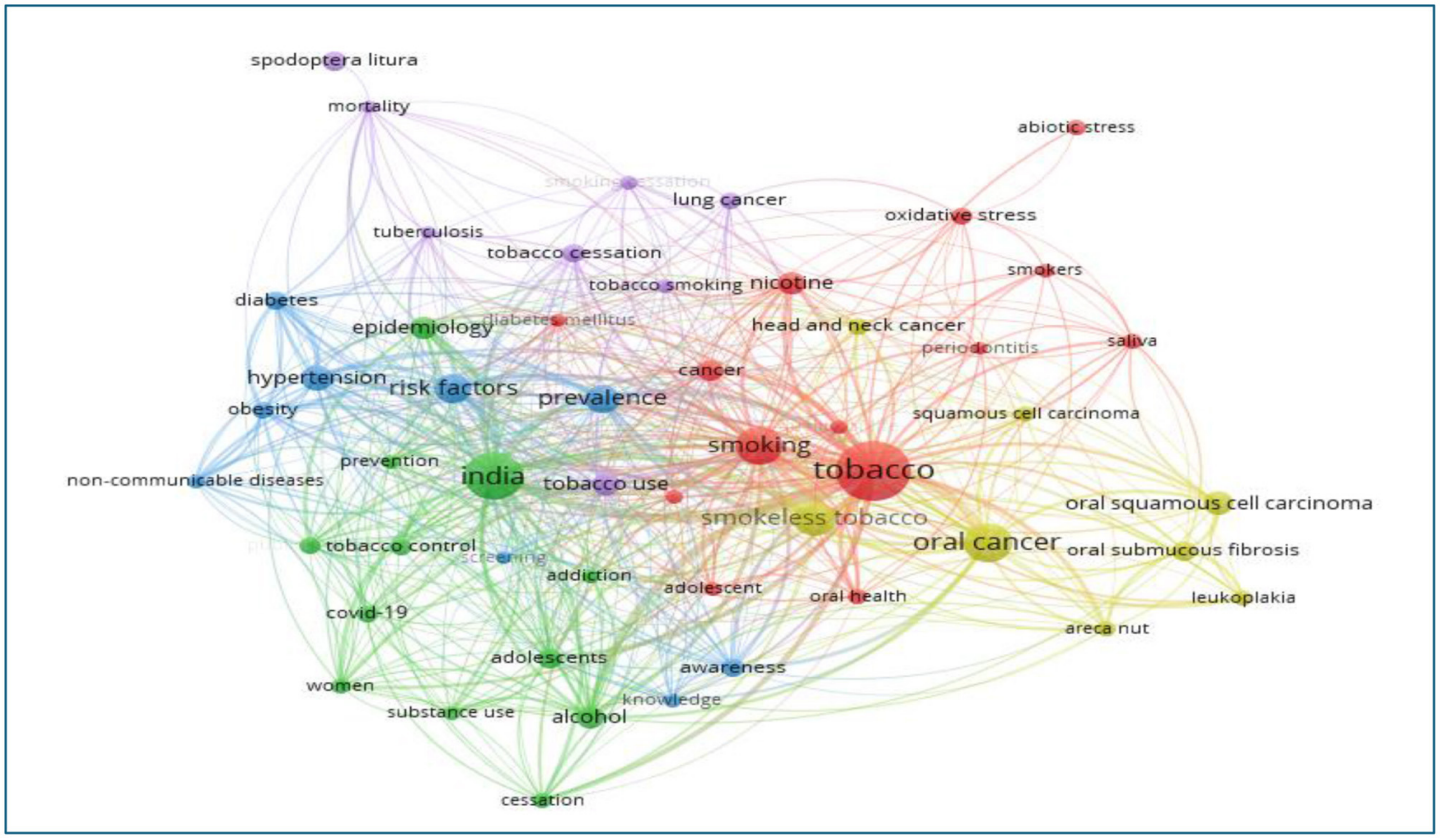
Network visualization map of author keywords co-occurrence in tobacco research publications from India (2003–2024).

Keywords are the core vocabulary of a paper, which succinctly summarize the main content and are also frequently used throughout the article. We extracted 51 out of 12,606 keywords with a frequency of 37 or more for co-occurrence analysis in Vos viewer to explore trends and hotspots in the field of research (Figure 13). The visual representations classify keywords into four clusters, as shown in Table 9. Table 10 highlights the progressive increase in the annual occurrence of the author's keywords related to tobacco research. It indicates the trending or popular research topics in the field, growing interest or emerging trends in that topic. This table also highlights the shifts in research focus.

**Table 9:** Details of keywords in different clusters of network co-occurrence of authors' keywords.

** Cluster 1 (14 items) red color **	** Cluster 2 (12 items) green color **	** Cluster 3 (9 items) blue color **	** Cluster 4 (8 items) yellow colored **	** Cluster 5 (8 items) purple colored **
Abiotic stress	Addiction	Awareness	Areca nut	Lung cancer
Adolescent	Adolescents	Diabetes	Head and neck cancer	Mortality
Cancer	Alcohol	Hypertension	Leucoplakia	Smoking cessation
Depression	Cessation	Knowledge	Oral cancer	Spodoptera literature
Diabetes mellitus	COVID-19	Non-communicable disease	Oral squamous cell ca	Tobacco cessation
Nicotine	Epidemiology	Obesity	Oral submucous fibrosis	Tobacco smoking
Oral health	India	Prevalence	Smokeless tobacco	Tobacco use
Oxidative stress	Prevention	Risk factors	Squamous cell carcinoma	Tuberculosis
Periodontitis	Public health	Screening
Quality of life	Substance use	
Saliva	Tobacco control	
Smokers	Women	
Smoking		
Tobacco		

**Table 10:** Word frequency over time in the field of tobacco research.

**Keyword**	**Occurrences**	**Total link strength**
Tobacco	727	824
India	453	558
Oral cancer	319	312
Smoking	299	446
Smokeless tobacco	239	265
Risk factors	182	224
Prevalence	159	209
Hypertension	133	179
Oral squamous cell carcinoma	122	80
Epidemiology	112	80
Alcohol	107	198

#### Trending topics

**Figure 14:**
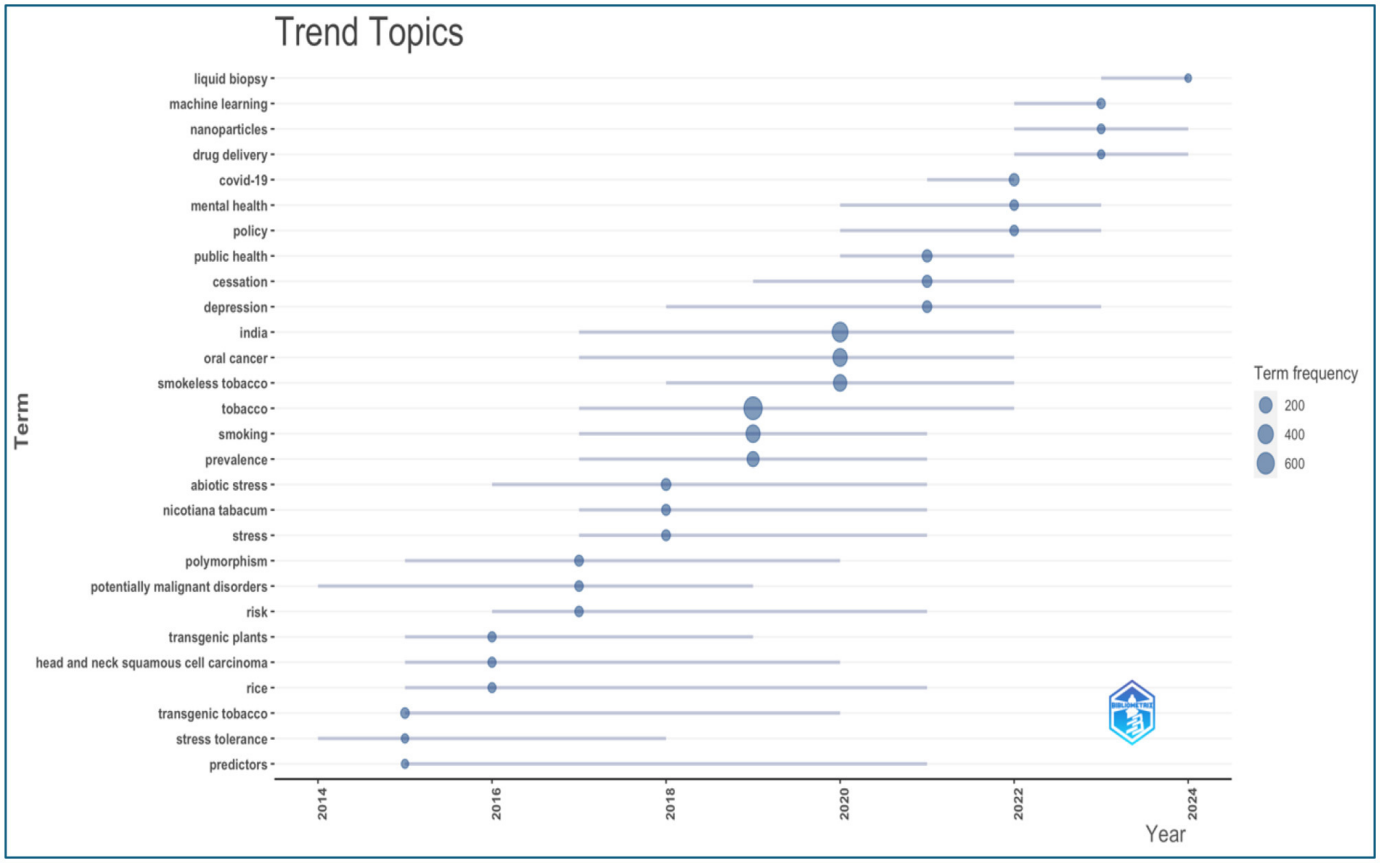
Trending topics in the tobacco research publications from India (2003–2024).

Since 2014, ~30 topics have shown notable increases in frequency. Figure 14 illustrates the occurrence trends, highlighting keywords such as “machine learning,” “nanoparticles,” “drug delivery,” “COVID-19,” “mental health,” “oral cancer,” and “smoking” as gaining prominence in recent years. This suggests that these research areas are currently receiving considerable attention and may reflect emerging trends in tobacco research.

## Discussion

This comprehensive bibliometric analysis reveals not only the expansion of national tobacco research over the past two decades but also several critical inflection points and persistent gaps.

Similar surges in tobacco research output have been reported in other low- and middle-income countries following FCTC ratification, suggesting a globally shared impetus toward tobacco control scholarship (Warner, 2005; Berg et al., 2018). The overwhelming dominance of medical and biomedical research (e.g., medicine, biochemistry, genetics) confirms earlier findings that tobacco-related morbidity remains the central concern driving publication trends (John, 2005; Pahari et al., 2023). However, the underrepresentation of social sciences, behavioral studies, and environmental research mirrors existing literature noting a fragmented understanding of the socio-cultural contexts of tobacco use (Claire et al., 2020; Parascandola et al., 2022). This suggests an ongoing disciplinary silencing of the “lived experience” of tobacco users, especially in rural or marginalized populations. An important gap observed in the analysis is the lack of explicit focus on smokeless tobacco, which constitutes a significant portion of tobacco use in India (Pahari et al., 2023). While some publications may have jointly addressed smoking and smokeless forms, this distinction is rarely made clear in bibliometric summaries or keyword analyses (Lahoti and Dixit, 2021). Given the high burden of disease from smokeless tobacco in India, particularly oral cancers and submucous fibrosis, future research efforts should disaggregate these categories to ensure more targeted prevention strategies (Gupta and Ray, 2003; Nair et al., 2004). Additionally, this study directly responds to longstanding gaps in tobacco control scholarship by illuminating the underrepresentation of research on the long-term health impacts of novel nicotine products and the socio-cultural dimensions of tobacco use in LMICs. Our analysis reveals that, while biomedical and clinical research dominates the field, studies examining the chronic effects of e-cigarettes and heated tobacco products are notably scarce. This is concerning given their rising popularity and ambiguous public health implications. Additionally, socio-behavioral research—particularly that which explores gender, caste, rurality, and poverty as drivers of tobacco initiation and persistence—remains peripheral in India's research ecosystem. By exposing these deficits, this study advocates for a broader epistemological shift that integrates cultural anthropology, behavioral science, and qualitative methodologies into mainstream tobacco research. Such an interdisciplinary pivot is essential not only for developing context-specific interventions but also for dismantling structural inequities in research agendas and funding allocations, especially in countries most vulnerable to the tobacco epidemic. Our analysis revealed limited representation of research on second-hand smoke exposure, environmental consequences of tobacco farming, and occupational exposure—all areas flagged by international experts as underexplored yet high-priority domains (Puljević et al., 2022). For example, Claire et al. (2020) have emphasized the urgent need to examine the broader ecological impacts of tobacco agriculture and waste (Claire et al., 2020). The observed institutional dominance of Indian organizations, particularly AIIMS and the Public Health Foundation of India, reflects India's high burden of tobacco-related disease, but also underscores the limited international collaboration visible in our co-authorship networks. This is consistent with previous critiques of South Asian tobacco research as “nationally siloed,” hindering cross-border learning (Berg et al., 2018; Meissner et al., 2022). While intra-national collaborations are strong, especially among urban academic centers, there is a pressing need to extend these networks to international and rural research institutions to support a more equitable and diverse research ecosystem (Lindson et al., 2017). Although the majority of funding stems from national organizations such as CSIR and ICMR, our study also detected contributions from international agencies like the National Institutes of Health (NIH) and the U.S. Department of Health and Human Services. However, detailed analysis of how these funds were allocated—whether directly to Indian institutions or through collaborative projects remains limited in bibliometric databases. This gap warrants further manual tracing through funding acknowledgments in primary articles to better understand global financial engagement in India's tobacco research landscape (Meissner et al., 2022). The prominent authors identified, such as Dr. Prakash C. Gupta and Dr. Dhirendra Sinha, have not only contributed prolifically but have also shaped the field's foundational literature, particularly in epidemiological surveillance and tobacco control program evaluation (Gupta and Ray, 2003; Rani et al., 2003). It is also evident that tobacco research in the reviewed literature is predominantly conducted by male authors, suggesting a gendered dimension in the production of knowledge within this field. This male-dominated authorship may influence the research priorities and perspectives represented in the literature, potentially overlooking gender-specific nuances in tobacco use and control. Additionally, the result highlights a clear preference for quantitative methodologies, with comparatively limited engagement with qualitative approaches. This methodological imbalance may constrain a deeper understanding of the social, cultural, and behavioral contexts of tobacco use, which are often better captured through qualitative inquiry. These observations warrant inclusion in the discussion section to underscore existing gaps and advocate for more inclusive and methodologically diverse research practices. Yet, the geographic clustering of these authors in urban India and the USA also reflects persistent regional disparities in research leadership and funding access, an issue echoed in previous bibliometric assessments of tobacco research in Asia (Ribisl, 2012; Shaikh et al., 2022). Journal analysis confirms the growing contribution of Indian public health journals, although publication in high-impact global journals remains limited. This may reflect not only language and resource barriers but also misalignment between international publication priorities and national health needs (KA et al., 1996). Efforts to enhance manuscript writing training and institutional support for early-career researchers could help bridge this gap. Co-citation and keyword analyses illuminate a recent methodological shift in the field. The integration of machine learning, nanotechnology, and interest in COVID-19-related respiratory impacts (Huang et al., 2024). The role of tobacco use in exacerbating COVID-19 outcomes has been explored in emerging research, which indicates that smokers and users of smokeless tobacco products may experience more severe pulmonary complications during viral infections (Claire et al., 2020). Additionally, COVID-19 heightened public and research awareness about lung health and vulnerability, further catalyzing investigations into the immunological and physiological overlap between tobacco-induced damage and viral pathogenesis. However, such findings are still in their early stages and demand more rigorous epidemiological validation. This study stands out for its novel integration of bibliometric analytics with public health priorities, offering a panoramic view of tobacco research in India from 2003 to 2024. Unlike prior research that has typically focused on isolated health outcomes or tobacco prevalence, this study distinguishes itself by mapping publication trends, institutional collaborations, keyword trajectories, and funding patterns—while also critically identifying neglected areas such as the long-term health effects of emerging nicotine products and the socio-cultural drivers of tobacco use. The potential audience includes public health researchers, policymakers, funding agencies, and global health organizations seeking to understand the evolution and direction of national tobacco control efforts. The added value lies in its ability to inform research prioritization and policy design by highlighting underrepresented themes like smokeless tobacco, rural health disparities, and gendered consumption patterns. Policy implications are profound: the study provides an empirical foundation for more equitable funding allocations, fosters interdisciplinary collaboration, and supports the development of context-specific tobacco control strategies that go beyond biomedical paradigms to address behavioral, environmental, and cultural determinants of use. This makes the study not only a methodological contribution to bibliometric science but also a strategic tool for shaping future public health interventions and policy frameworks. In summary, while the volume and visibility of tobacco research in India have grown considerably, the field remains medically dominated, urban-centric, and under-integrated with behavioral, environmental, and international research. Bridging these disciplinary and geographic gaps will require both structural investment and intentional collaboration strategies (Hicks et al., 2019; Claire et al., 2020; Puljević et al., 2022). In conclusion, the study reveals that tobacco research in India has expanded significantly over the past two decades, primarily driven by public health priorities and evolving nicotine product landscapes. There has been substantial output in medical and biomedical domains, reflecting concerns around tobacco-induced morbidity and mortality.

However, several critical research gaps remain.

These include:

1. Insufficient disaggregation between smoking and smokeless tobacco in empirical analyses,
2. Underrepresentation of environmental and socio-behavioral dimensions of tobacco use,
3. Limited studies on second-hand smoke exposure and occupational hazards related to tobacco cultivation, and
4. Inadequate exploration of international funding streams and collaborations in published work.

Furthermore, the effects of tobacco use on COVID-19 outcomes require deeper investigation, particularly regarding respiratory comorbidities and differential risks among tobacco users.

Addressing these gaps requires a broader interdisciplinary approach, enhanced international partnerships, and targeted funding mechanisms that prioritize equity and underserved research areas.

## Limitations

There are several limitations to note in this study. Firstly, we only utilized SCOPUS as our database, which may have led to the omission of relevant papers from other databases. Secondly, there is a possibility that significant non-English papers were overlooked, resulting in research bias and a reduction in credibility. Finally, due to the constant database updates, recently published high-quality articles may have been underestimated because of their inadequate citations.

## Data Availability

The original contributions presented in the study are included in the article/supplementary material, further inquiries can be directed to the corresponding author.
